# A Rapid Immunochromatographic Method Based on a Secondary Antibody-Labelled Magnetic Nanoprobe for the Detection of Hepatitis B preS2 Surface Antigen

**DOI:** 10.3390/bios10110161

**Published:** 2020-10-31

**Authors:** Yangyang Cai, Jun Yan, Li Zhu, Hengliang Wang, Ying Lu

**Affiliations:** 1 College of Food Science and Technology, Shanghai Ocean University, Shanghai 201306, China; m170200408@st.shou.edu.cn (Y.C.); jyan@shou.edu.cn (J.Y.); 2 Laboratory of Quality & Safety Risk Assessment for Aquatic Products on Storage and Preservation (Shanghai), Ministry of Agriculture, Shanghai 201306, China; 3 Shanghai Engineering Research Center of Aquatic-Product Processing and Preservation, Shanghai 201306, China; 4 Beijing Institute of Biotechnology, Beijing 100071, China; jewly54@bmi.ac.cn (L.Z.); wanghl@bmi.ac.cn (H.W.)

**Keywords:** hepatitis B, hepatitis B preS2 antigen, magnetic nanoparticles, immunochromatographic assay, rapid detection

## Abstract

Hepatitis B is a globally prevalent viral infectious disease caused by the hepatitis B virus (HBV). In this study, an immunochromatographic assay (ICA) for the rapid detection of hepatitis B preS2 antigen (preS2Ag) was established. The magnetic nanoparticles (MNPs) indirectly labelled with goat anti-mouse (GAM) secondary antibody were applied as a nanoprobe for free preS2 antibody (preS2Ab) capturing and signal amplification. By employing sample pre-incubation processing as well, preS2Ag-preS2Ab was sufficiently caught by the GAM-MNPs probe in 5 min. A qualitative sensitivity of 625 ng/mL was obtained by naked-eye observation within 15–20 min. A standard curve (0–5000 ng/mL) was established, with a quantitative limit of detection (LOD) of 3.6 ng/mL, based on the stability and penetrability of the magnetic signal characteristics. The proposed method for preS2Ag was rapid (~25 min, cf. ELISA ~4 h) and had a good accuracy, which was verified using an ELISA kit (relative error < 15%). Large equipment and skilled technicians were not required. The sensitivity and specificity of the developed GAM-MNPs-ICA method were 93.3% and 90% in clinical serum samples (n = 25), respectively. A good detection consistency (84%) was observed between the developed ICA method and 2 types of commercial ELISA kits, indicating that the GAM-MNPs-ICA has a potential application in large-scale screening for and point-of-care diagnosis of hepatitis B or other infectious diseases.

## 1. Introduction

Hepatitis B remains a great threat to public health. It is estimated that more than 257 million people have suffered from chronic hepatitis B infection worldwide, and almost 9 million people have died from liver failure, cirrhosis, hepatocellular carcinoma, and other related diseases caused by HBV [1]. Hepatitis B surface antigen (HBsAg) is the envelope protein of HBV, which is the most conventional serological marker for the screening, diagnosis, and antiviral therapy evaluation of hepatitis B. The overexpression (10–100,000 folds excess) of HBV genome-free subviral particles (SVPs) was found in acute and chronic hepatitis B patients [2], making HBsAg an easily detected biomarker. Commercial HBsAg serological tests for HBV include radioimmunoassay (RIA), enzyme-linked immunosorbent assay (ELISA), chemiluminescence immunoassay (CLIA), and electrochemiluminescence immunoassay assay (ECLIA). However, these methods are complex, time consuming, and require large equipment and professional technicians. Additionally, given the complex and dynamic variations of HBV, a single HBsAg test has drawbacks in relation to occult HBV infection (OBI) identification and antiviral therapy evaluation, and further confirmation of other biomarkers is required [3].

The HBsAg consists of three components: preS1, preS2, and S proteins, among which the S protein is the main target of commercial detection kits. In recent years, in order to develop novel markers complementary to the traditional S protein, the clinical values and biological roles of preS1Ag, preS2Ag, and their complex proteins, including large surface protein (L protein, preS1+preS2+S) and middle surface protein (M protein, preS2+S), were studied and evaluated [4,5,6,7].

The preS2Ag is located on the surface of HBV mature virions and SVPs. It has a transcriptional transactivator function [8] and contains binding sites for fibronectin, transferrin, and polymerized human serum albumin (pHSA) [9], which are associated with virus entry, release, and active replication. Besides, some studies have shown that the amount and proportions of HBsAg components could be used to identify inactive HBV carriers [10], OBI [11], and predict the response to antiviral therapy [12]. Considering the fact that there have been few reports on clinical preS2Ag detection, it is essential to develop a rapid and quantitative method for preS2Ag.

ICA, also known as lateral flow immunoassay (LFIA), is the most popular point-of-care diagnosis tool in many fields. ICA has been widely applied in HBV screenings, such as self-examinations, health checks, blood donations, emergencies, and epidemiological investigations, due to its great advantages, including a simple operation, low cost, and short analysis time [13]. Traditional ICA products employ colored or luminescent nanoparticles as detection probes. However, the quantitative detection may be influenced by the color of the sample matrix, light reflection and scattering of the membrane, and the optical signals for observation and further quantitative measurement exist only within a thickness of 10–20 μm on the surface of the nitrocellulose (NC) membrane, resulting in 90% signals being undetectable [14]. MNPs are another widely used detection probe and have shown great potential in ICA research due to their chemical stability, easy labelling operation, and low background interference. Additionally, magnetic signals can be totally collected from the surface to the inside (100 μm) of the NC membrane by a magnetic assay reader to achieve a more sensitive and accurate quantitative analysis [15]. Recently, MNPs-based ICA methods have been applied in the detection of drugs [16], toxins [17], biochemical markers [18,19], bacteria [20], and viruses [21].

In this work, a quantitative ICA method based on indirectly labelled GAM-MNPs were developed for rapid preS2Ag detection. A sample pre-incubation procedure and indirect labelling strategy were introduced to help reduce the usage of primary antibody, while maintaining the bioactivity of the antibody, and ensure a sufficient combination of antigen and antibody to capture a trace amount of preS2Ag [22]. MNPs were applied as a quantitative probe, instead of colloidal gold, to achieve a more accurate and rapid quantitative detection by utilizing their sensitive and stable magnetic signal.

## 2. Materials and Methods

### 2.1. Materials

The preS2Ag peptides, CMQWNSTAFHQALQDPRVRGLYFPAGGAA (120–145 amino acids with cysteine modified, UniProtKB-Q6VBP1), were synthesized by Genscript Co., Ltd. (Shanghai, China). The anti-preS2 antibody was purchased from Abcam plc. (Cambridge, UK). The MNPs were purchased from Allrunnano Co., Ltd. (Shanghai, China). The goat anti-mouse (GAM) secondary antibody, NC membrane (CN 140), sample pad, conjunction pad, absorbent pad, and polyvinyl chloride (PVC) base pad were purchased from Jiening Biotech Co., Ltd. (Shanghai, China). The mouse IgG was purchased from Solarbio Science & Technology Co., Ltd. (Beijing, China). The bovine serum albumin (BSA) was purchased from Sigma-Aldrich (St. Louis, MO, USA). The 4-(*N*-maleimidomethyl) cyclohexane-1-carboxylic acid succinimide ester (SMCC) was purchased from Thermo Fisher Co., Ltd. (Waltham, USA). The EDC (1-ethyl-(3-dimethylaminopropyl) carbonyl diimide hydrochloride), *N*-hydroxysuccinimide (NHS), 2-morpholineethanesulfonic acid (MES), Tween-20, sodium azide, and BCA protein quantitative kit were purchased from Sangon Biotech Shanghai Co., Ltd. (Shanghai, China). The disodium hydrogen phosphate, sodium dihydrogen phosphate, boric acid, and sodium tetraborate were purchased from Sinopharm Chemical Reagent Co., Ltd. (Shanghai, China). The full-length hepatitis B preS2Ag and ELISA kit for preS2Ag were obtained from Cusabio Co., Ltd. (Wuhan, China). The hepatitis B preS1Ag, HBsAg, hepatitis B e antigen (HBeAg), HIV surface antigen, and ELISA kit for HBsAg were obtained from Kehua Bio-Engineering Co., Ltd. (Shanghai, China).

### 2.2. Preparation of preS2Ag-BSA Conjugates

The conjugate of preS2Ag and BSA (preS2Ag-BSA) was prepared using the SMCC method. First, 200 μL of SMCC (1 mg/mL) was reacted with 1 mL of BSA (12 mg/mL) in a vertical mixer wrapped in tin foil for 30 min. The reaction solution was added to a desalted column, which was pre-washed with 10 mM PBS buffer 4 times. Next, 1.3 mL of PBS was added after the sample entered the packed bed completely. Subsequently, the column was eluted with 3.5 mL of PBS, and the eluate was collected. Finally, 200 μL of preS2Ag (1 mg/mL) was reacted with 200 μL of eluate at 25 °C overnight. The microplate reader was employed to measure the UV absorption spectra, and SDS-PAGE was utilized to examine the molecular weights of preS2Ag, BSA, and their conjugate.

### 2.3. Preparation of GAM-MNPs

The NHS/EDC chemical coupling method was used for the GAM-MNPs preparation, according to the method of Du et al. [23]. First, 1 mg of MNPs was reacted with 2.5 mg of NHS and EDC in 500 μL of the MEST buffer (pH 5.0, 10 mM MES, with 0.05% Tween-20 (*v*/*v*)) for 30 min at 25 °C. After being washed twice with 500 μL of BST buffer (pH 9.0, 5 mM borate buffer (BS), with 0.05% Tween-20 (*v*/*v*)), different amounts of GAM antibody were added and reacted for 3 h. After being washed with 500 μL of BST buffer three times, 500 μL of 10 mg/mL of BSA was added and reacted for 30 min at 25 °C. Finally, the obtained GAM-MNPs were stored in the BST buffer with 0.5 mg/mL of NaN_3_ and 1 mg/mL of BSA at 4 °C. The amount of GAM coupled with MNPs and the coupling ratio were determined by a BCA protein quantitative kit.

### 2.4. Preparation of GAM-MNPs-ICA Test Strips

The construction of the test strips was based on the method of Yan [24] et al., with some modifications. The preS2Ag-BSA conjugate was sprayed with a dispensing instrument (XYZ3020, Jiening Biotech Co., Ltd., Shanghai, China) on the surface of the NC membrane as a test line (T-line), and 1.5 mg/mL of mouse IgG was sprayed as a control line (C-line). After drying at 37 °C for 2 h, the sample pad, conjugate pad, and absorbent pad were pasted onto the PVC base pad in sequence. Finally, the assembled composites were cut into strips with a width of 5 mm and preserved in aluminum foil bags.

### 2.5. Detection Procedure of GAM-MNPs-ICA

Firstly, 50 μL of the sample was incubated with 50 μL of preS2Ab and 8 μL of GAM-MNPs for 10 min to form the GAM-MNPs-preS2Ab-preS2Ag complexes. Subsequently, the sample mixed with 15 μL of chromatographic solution (BS buffer with 0.2 g/mL BSA and 5% Tween-20(*v*/*v*)) was added to the sample pad of the test strip. The qualitative test results could be obtained by the naked eye within 15–20 min, and the quantitative results for the T and C lines were measured by a magnetic reader. The *B*/*B*_0_ ratio between the sample (*B*) and negative control (*B*_0_) was calculated as an index for the quantification analysis [25] according to the following equation:

(1) $$B/B_{0}=\frac{MS_{T}/MS_{C}}{MS_{T_{0}}/MS_{C_{0}}}$$

where *B* is the ratio of the magnetic signals of the *T* and *C* lines of sample; *B*_0_ represents the ratio of the magnetic signals of the *T* and *C* lines of the negative control; $MS_{T}$ and $MS_{C}$ are the magnetic signals of the *T* and *C* lines of the sample, respectively; and $MS_{T_{0}}\;\mathrm{and}\;MS_{C_{0}}$ are the magnetic signals of the *T* and *C* lines of the negative control, respectively. A high value for *B*/*B*_0_ indicated a strong positive result.

### 2.6. Detection Condition Optimization of GAM-MNPs-ICA

In order to improve the detection performance of the test strip, the preS2Ag-BSA concentration on *T*-line (0.125, 0.25, 0.5, and 1 mg/mL), the amount of GAM secondary antibody coupled with MNPs (10, 20, 40, 80, 120, and 160 μg), and the incubation concentration of preS2Ab (12.5, 25, 50, 100, 200, and 400 ng/mL) were optimized. The optimization results were evaluated using samples with 0, 50, 500, and 5000 ng/mL of preS2Ag spiked in PBS to choose the appropriate line color and *B*/*B*_0_ ratios.

### 2.7. Detection Performance Evaluation

#### 2.7.1. Sensitivity

A serial dilution of preS2Ag (9.8–5000 ng/mL) in PBS was used to establish a standard curve. The qualitative sensitivity was defined as the concentration when the T-line became invisible. The quantitative LOD was calculated as the main signals plus a three-fold standard deviation of the negative samples [26].

#### 2.7.2. Specificity

Hepatitis B preS2Ag, preS1Ag, HBsAg, HBeAg, and HIV surface antigen were individually spiked in PBS (10 μg/mL) and used as test samples for specificity evaluation.

#### 2.7.3. Accuracy Validation

Positive samples with different concentrations of preS2Ag (5, 50, 500, and 5000 ng/mL) were prepared by diluting them in 10% Hepatitis negative human serum (1:10, *v*/*v*). Moreover, the 10% Hepatitis negative human serum was used as a negative control. The prepared samples were tested by the developed GAM-MNPs-ICA and commercial ELISA kit. The concentrations of each sample were calculated and analyzed according to the established quantitative standard curves.

#### 2.7.4. Stability

The storage stability of GAM-MNPs-ICA was evaluated by the accelerated experiment proposed by Liu et al. [27]. Four groups of samples with 0, 50, 500, and 5000 ng/mL of preS2Ag spiked in PBS were used as test samples. The tested strips were placed in an oven at 60 °C for 6 d, and the magnetic signals were repeatedly measured every day.

#### 2.7.5. Reproducibility

Six groups of test strips from the same batch and different batches were tested with 0, 50, 500, and 5000 ng/mL of preS2Ag spiked samples. The intra- and inter-batch values were calculated and compared.

### 2.8. Clinical Sample Analysis

In total, 25 clinical samples were kindly provided by the Shanghai Sixth People’s Hospital East (Shanghai, China) and stored at −80 °C for subsequent study. HBsAg assays with the commercial ELISA kit were adopted to identify HBsAg positive and negative samples. Each sample was individually numbered and tested by GAM-MNPs-ICA and another ELISA kit for preS2Ag.

## 3. Results

### 3.1. Detection Principle of GAM-MNPs-ICA

**Figure 1:**
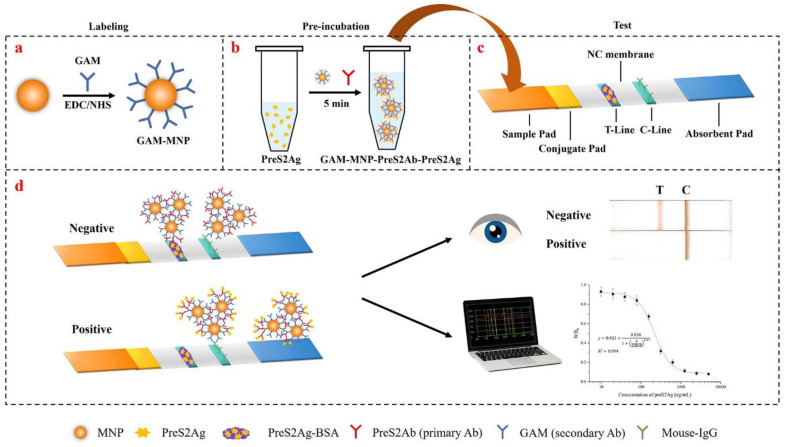
Schematic illustration of GAM-MNPs-ICA for preS2Ag detection. (**a**) Preparation of the GAM-MNP probe using the EDC/NHS method. (**b**) Pre-incubation of preS2Ag, preS2Ab, and GAM-MNPs. (**c**) Structure of the test strip. (**d**) Judgment of the negative and positive results and qualitative and quantitative analysis methods.

The developed GAM-MNPs-ICA was composed of a sample pre-incubation procedure and competitive ICA format (Figure 1). First, GAM-MNPs probes were prepared by coupling GAM secondary antibody onto the surface of MNPs using the EDC/NHS coupling method (Figure 1a). In order to ensure a sufficient combination of antigen and antibody and capture a trace amount of preS2Ag, a sample pre-incubation of preS2Ag, preS2Ab, and GAM-MNPs probes was then applied to form a complex of GAM-MNPs-preS2Ab-preS2Ag (Figure 1b). The residual preS2Ab on the GAM-MNPs that was not combined with preS2Ag in the sample could be captured by the preS2Ag-BSA at the T-line. Moreover, the preS2Ab on the complexes could react with mobilized mouse IgG to form a visible C-line. Based on the competitive principle, the more free preS2Ag in the sample, the less preS2Ab bound at the T-line. Therefore, when the color of the T-line was obviously lighter than the negative sample, or even disappeared, it could be considered as a positive result. When the color of the T-line was close to or deeper than the negative sample, it could be judged as a negative result. Finally, the magnetic signals of the T and C lines could be observed by the naked eye and quantified by a magnetic assay reader (Figure 1d).

It was reported that the indirect type of ICA using secondary antibody labelling was more sensitive than direct ICA based on primary antibody labelling [28], given that the indirectly labelled probes and primary antibody could be added to the sample pre-incubation step and intergraded in the sample dilution procedure to ensure a sufficient antigen–antibody reaction in the solution environment. Besides, the secondary antibodies on different MNPs may capture the same primary antibody and agglomerate to construct a network structure, thus effectively amplifying the specific signals. Additionally, the application of MNPs was conducive to achieving a more accurate quantification and improving the detection performance [29].

### 3.2. Characterization of Conjugated Antigen

**Figure 2:**
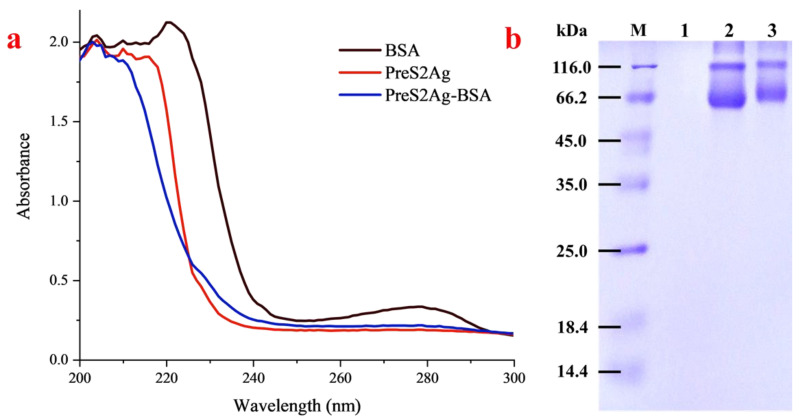
Characterization of conjugated antigen. (**a**) UV absorption spectrum. (**b**) SDS-PAGE. M: Marker; 1: preS2Ag; 2: BSA; 3: preS2Ag-BSA.

In this study, the synthesized preS2Ag peptides were pre-modified with cysteine to provide active sulfhydryl groups, and preS2Ag-BSA conjugates were obtained using the SMCC coupling method. As shown in Figure 2a, the UV absorption wave patterns and absorption peaks of the preS2Ag-BSA conjugate in the range of 200–240 nm were different from those of the BSA and unconjugated preS2Ag, thus showing a successful coupling reaction. Correspondingly, it was observed that the band of the preS2Ag-BSA in SDS-PAGE moved more slowly compared with BSA (Figure 2b), indicating that the molecular weight increased after coupling and confirming the coupling of preS2Ag and BSA. Similar migration rate changes of the conjugate in SDS-PAGE analysis were reported by Shen et al. [30]. The unconjugated preS2Ag did not show any band, because the molecular weight was too small (3.3 kD) to remain in the gel.

### 3.3. Optimization of Detection Conditions

#### 3.3.1. Optimization of the T-line Concentration

**Figure 3:**
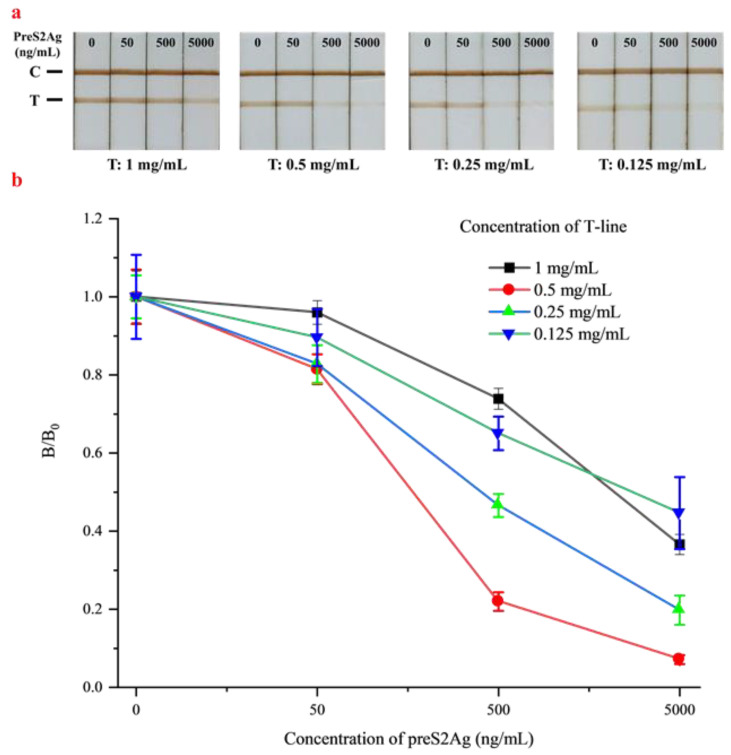
Optimization of different T-line concentrations with 0, 50, 500, and 5000 ng/mL of preS2Ag. (**a**) Qualitative results. (**b**) Quantitative results. The error bars represented the standard deviation of three repeats (n = 3).

The optimization results of the preS2Ag-BSA concentration at the T-line are shown in Figure 3. For a competitive ICA, the stronger the positive reaction, the lighter the T-line (which can also be colorless). Obviously, the color of the T-lines in the same group became lighter with the increasing concentration of preS2Ag in the sample, while the test strips with the addition of 500 and 5000 ng/mL were almost invisible when the coated preS2Ag-BSA concentration was 0.5 mg/mL or lower (Figure 3a). It was found that the *B*/*B*_0_ ratios of the T-line changed most significantly at 0.5 mg/mL of preS2Ag-BSA (Figure 3b). A lower value of *B*/*B*_0_ indicated a stronger difference in the magnetic signals between the sample and negative control. Thus, a wider range of *B*/*B*_0_ demonstrated a better competition relationship, which was more conducive to the establishment of a sensitive detection method. Similar results were reported in the study on the ractopamine test strip by Hu [31]. Thus, 0.5 mg/mL was considered as the optimal concentration of preS2Ag-BSA coated at the T-line.

#### 3.3.2. Optimization of the GAM Amount Coupled with MNPs

**Figure 4:**
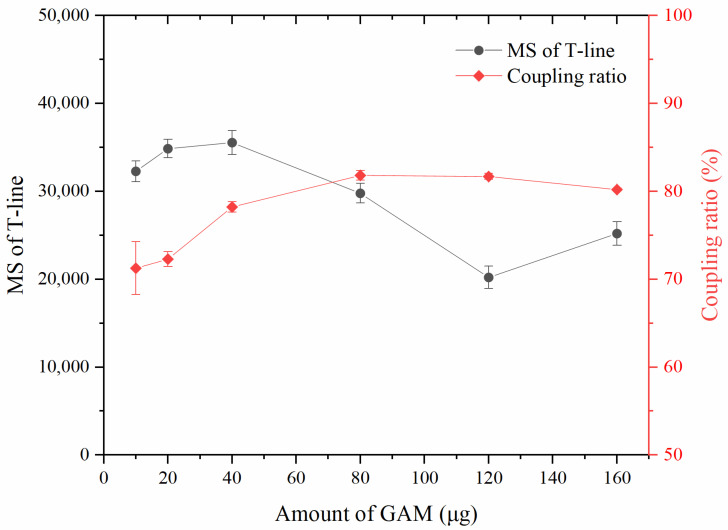
Optimization of the secondary antibody amount coupled onto the surface of MNPs. The error bars represent the standard deviation of three repeats (n = 3).

In this study, the GAM-MNPs probe was designed to capture the free preS2Ab and complex of preS2Ab-preS2Ag, and an appropriate amount of GAM coupled on MNPs helped to form a clear line color, while minimizing the nonspecific coloration. As presented in Figure 4, the magnetic signals of the T-line increased gradually with the increasing of the GAM amount. They reached their highest point at 40 μg and then subsequently decreased with the increasing of the GAM amount. The decrease was probably due to the agglomerated GAM-MNPs, because excessive secondary antibody forms a large network structure with primary antibody and jams in the NC membrane, thus reducing the valid combination of GAM, preS2Ab, and preS2Ag. A rebound at 160 μg may be due to the excess formation of large complexes of GAM-preS2Ab, erroneously magnifying the nonspecific signals. The coupling ratios gradually increased at first and reached their highest point at 80 μg. Then, they sightly decreased with the continued increase of the GAM amount. The ratios were all above 70%, thus proving the efficient coupling of GAM and MNPs using the EDC/NHS method. The optimal amount of GAM for the preparation of the GAM-MNPs probe was considered to be 40 μg because of the high magnetic signals and acceptable coupling ratio.

#### 3.3.3. Optimization of the preS2Ab Concentration in the Pre-Incubation Solution

**Figure 5:**
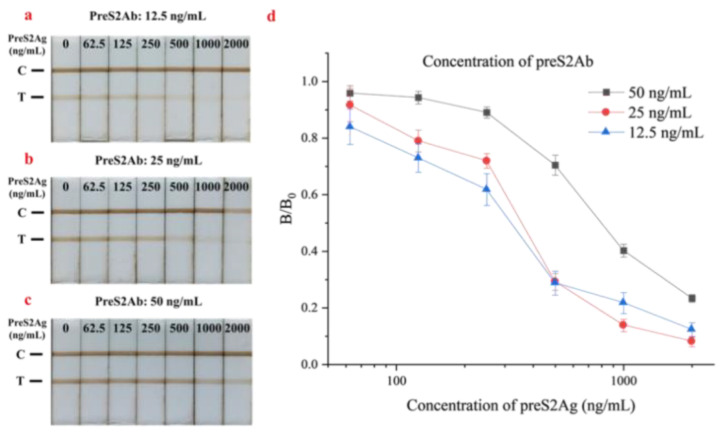
Qualitative results (**a**–**c**) and quantitative results (**d**) for different concentrations of preS2Ab antibody in sample pre-incubation. The error bars represent the standard deviation of three repeats (n = 3).

The concentration of primary antibody is closely related to the color intensity at the T-line of the test strip and greatly affects the detection performance. Typically, the higher the antibody concentration, the stronger the ability to bind antigen. Thus, the color of the T-line would be enhanced, while the color of the C-line would correspondingly be weakened. As a result, the T/C ratio rose, which was more conducive to observation and quantification for a competitive detection format. In order to compare the competitive efficiency, the preS2Ab of 12.5, 25, and 50 ng/mL were pre-incubated with preS2Ag of different concentrations (0, 50, 500, and 5000 ng/mL). The qualitative and quantitative results for preS2Ab optimization are demonstrated and plotted in Figure 5.

A lower concentration of primary antibody helped to form a competitive relationship with antigens in the sample and effectively improved the sensitivity. However, insufficient antibody lead to the weak color of the T-line and strengthened the nonspecific absorption [32]. The quantitative analysis curve showed that when the preS2Ab antibody concentration was 25 ng/mL, the *B*/*B*_0_ values for the maximum and minimum preS2Ag concentration varied most significantly (Δ*B*/*B*_0_ = 0.836). Moreover, the *B*/*B*_0_ ranges of the lower (12.5 ng/mL, Δ*B*/*B*_0_ = 0.717) and higher preS2Ab concentration (50 ng/mL, Δ*B*/*B*_0_ = 0.725) were inferior to that of the 25 ng/mL group. Thus, 25 ng/mL was selected as the optimal preS2Ab concentration for subsequent experiments.

### 3.4. Performance Evaluation of GAM-MNPs-ICA

#### 3.4.1. Sensitivity

**Figure 6:**
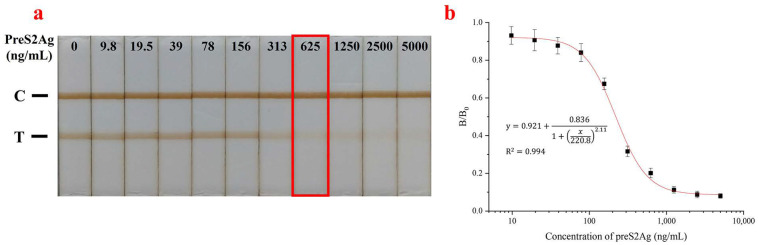
Detection sensitivity of the GAM-MNPs-ICA for preS2Ag detection. (**a**) Qualitative detection results. (**b**) Quantitative standard curve. The error bars represent the standard deviation of three repeats (n = 3).

The qualitative results and quantitative curve of the GAM-MNPs-ICA tested with a series of diluted preS2Ag are shown in Figure 6. It was observed that the T-line gradually grew fainter as the preS2Ag concentration increased, showing a typical change pattern of competitive ICA. The color of the T-line was almost invisible at 625 ng/mL, which was determined to be a qualitative limit (Red rectangular box) (Figure 6a). The quantitative concentration range spanned 0–5000 ng/mL (Figure 6b), and the correlation was good (R^2^ = 0.994). The quantitative sensitivity was calculated to be 3.6 ng/mL, which was enough for HBV detection due to the high-level overexpression of SVPs [33].

#### 3.4.2. Specificity

**Figure 7:**
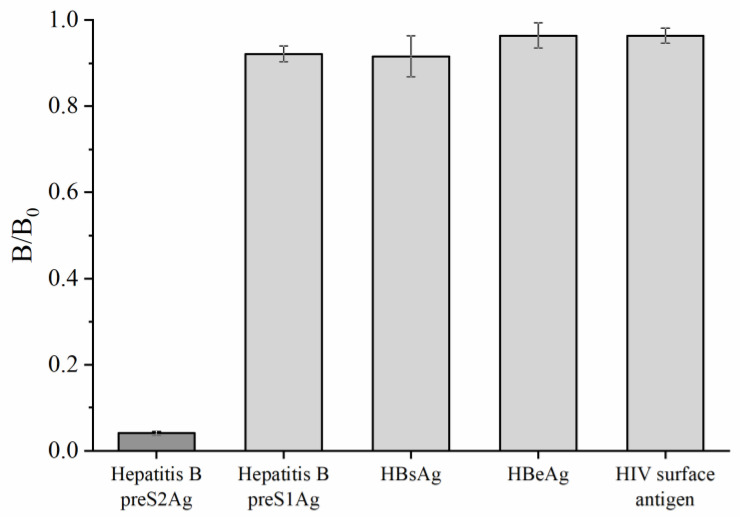
Quantitative results of the specificity analysis. The error bars represent the standard deviation of three repeats (n = 3).

Three HBV markers and HIV surface antigen were used to evaluate the specificity of GAM-MNPs-ICA. As illustrated in Figure 7, the *B*/*B*_0_ ratio of Hepatitis B preS2Ag was less than 0.1, implying a strong positive result, while the ratios of Hepatitis B preS1Ag, HBsAg, HBeAg, and HIV surface antigen were all close to 1.0, indicating negative results. Consequently, the developed GAM-MNPs-ICA was specific to preS2Ag.

#### 3.4.3. Accuracy Validation

A commercial ELISA kit against preS2Ag was employed to evaluate the accuracy of GAM-MNPs-ICA. As shown in Table 1, for serum spiked with different concentrations of preS2Ag (5, 50, 500, and 5000 ng/mL), the relative error of both methods was less than 15%, indicating a good consistency between the GAM-MNPs-ICA and ELISA kit. As the assay procedure of GAM-MNPs-ICA was greatly simplified (~25 min, cf. ELISA ~4 h), it might have a potential application prospect in the clinical analysis of Hepatitis B.

#### 3.4.4. Stability

**Figure 8:**
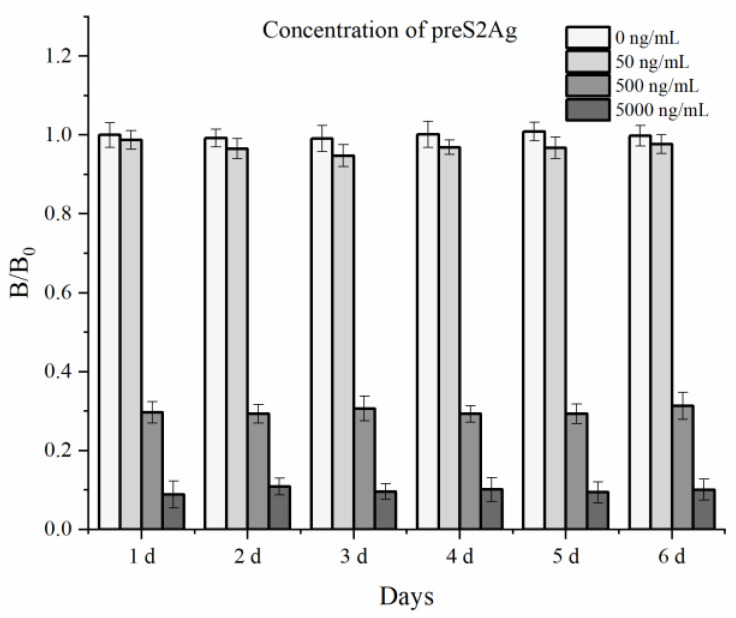
Stability evaluation of GAM-MNPs-ICA. The error bars represent the standard deviation of three repeats (n = 3).

The stability of GAM-MNPs-ICA was evaluated using a 6d accelerated experiment (Figure 8). Slight changes (CV < 7%) were found in the *B*/*B*_0_ ratios of different preS2Ag concentrations over 6 days, showing that the magnetic signals on the test strips were stable. According to the Arrhenius equation, one day of storage at 60 °C is equivalent to one month at 25 °C in a dry environment [34]. Thus, it could be preliminarily estimated that the GAM-MNPs-ICA test strips could be stored for at least 6 months at 25 °C, ensuring that the recheck results would not be affected by magnetic signal changes during preservation.

#### 3.4.5. Reproducibility

The evaluation of test strips of the same batch and different batches is shown in Table 2. It was denoted that the prepared test strips had a good reproducibility (CV < 15%) in the intra- and inter-assay, thus improving the test performance and reducing the detection error. Furthermore, the CV in the inter-assay was obviously higher than that in the intra-assay, suggesting that when performing a standard curve for quantitative calculations, the same batch of test strip products was required to ensure reliability. Interestingly, a higher concentration of preS2Ag brought about a larger CV, since fewer MNPs were captured by the T-line for measurement, and the background noises had a more obvious impact. Therefore, before testing high-concentration samples, an appropriate dilution should be performed to enhance the T-line signals, so that the *B*/*B*_0_ values would fall within a more accurate quantitative range to be analyzed.

### 3.5. Clinical Sample Analysis

HBsAg is the principal indicator in HBV infection. In this study, a commercial ELISA kit against HBsAg (Kit 2) was used to examine 25 serum samples, where 15 HBsAg positive samples and 10 negative samples were identified. Subsequently, the 25 samples were separately tested using the developed GAM-MNPs-ICA method and a commercial ELISA kit against preS2Ag (Kit 1). The number of true positives (TPs), false positives (FPs), false negatives (FNs), and true negatives (TNs) were recorded and are listed in Table 3. The sensitivity (TP/(TP+FN)) and specificity (TN/(FP+TN)) were calculated according to the description of Barnett et al. [35]. It was found that the detection results of 21 samples were the same for three methods, and the consistency was 84%. Among them, 12 serum samples were positive, while 9 samples were negative. According to the calculation results, the sensitivity and specificity were 93.3% and 90%, respectively, for the ICA method and 86.7% and 100%, respectively, for Kit 1. Thus, the developed ICA method was potentially applicable for the detection of preS2Ag in clinical serum samples. Among the 15 positive samples, only three samples were detected using GAM-MNPs-ICA or Kit 1, and one false positive result was found using the GAM-MNPs-ICA method in 10 negative samples. Since the genotypes of HBV, courses of HBV infection, mutants of HBsAg, and matrix effects of the serum sample may influence the detection results, further study was necessary to ensure the application possibility of GAM-MNPs-ICA.

## 4. Discussion

Hepatitis B infection accounts for more than 50% of hepatocellular carcinoma cases worldwide [36]. Since chronic HBV infection may not present obvious clinical symptoms, effective early diagnosis tools are of great significance for the control and management of hepatitis B. The HBsAg, HBsAb, HBeAb, HBeAg, and HBcAb are general serologic markers for HBV detection, and HBsAg is the most widely accepted hallmark in the evaluation of HBV infection and antiviral therapy response. Most researches on HBsAg focused on the function of the small surface protein of HBsAg. PreS antigen (preS1Ag+preS2Ag) is the *N*-terminal extension of small surface protein, and the preS antigen, as well as middle and large surface proteins, have gained widespread attention in recent studies on the role of oncogenic risk factors [6], identification of inactive HBV carriers [10], prognosis of therapeutic response [12], and prediction value of HBV infection phases and virological response [37].

Conventional immunoassays on hepatitis serological markers are cumbersome and time-consuming (~4 h) and require large equipment and skilled technicians. Thus, novel immunosensors were developed to simplify the operation and reduce costs. A comparison of several immunosensors for the quantitative detection of HBV serologic markers is summarized in Table 4. Obviously, ICA methods with various nanoparticle labels (colored, luminescent, and magnetic nanoparticles) have been widely applied in the quantitative detection of hepatitis B serological markers, with a short assay time (10~25 min), convenient operation, and good sensitivity [38,39,40,41,42]. However, clinical human serum samples have not been evaluated in the reported ICA methods [38,39,40,41], and other serologic markers, except HBsAg, were less reported. An electrochemical method based on an AuNPs-modified glassy carbon electrode was developed to detect preS1Ag; however, it took about 80 min [43].

In this study, a simple and rapid MNPs-ICA method for preS2Ag quantification was established. The indirect labelling of MNPs with secondary antibody was applied to capture free preS2Ab and amplify signals. Moreover, a 5-min sample pre-incubation procedure was introduced to maintain the maximum bioactivity of antibodies and fully ensure the reaction between antigen and antibodies. The assay time and complexity did not increase, as the pre-incubation could be completed during the sample dilution. The quantitative LOD of the proposed ICA method was 3.6 ng/mL, while the visual qualitative sensitivity was 625 ng/mL. The validation results using the commercial ELISA kits indicated that the assay procedure of GAM-MNPs-ICA was greatly simplified (~25 min, cf. ELISA ~4h). Additionally, the sensitivity and specificity of the developed GAM-MNPs-ICA method were 93.3% and 90% in clinical serum samples (n = 25), respectively. It was potentially applicable for detection of preS2Ag in clinical serum samples. Overall, the GAM-MNPs-ICA was rapid and sensitive, and the preS2Ag assay had a good correlation with the HBsAg assay, indicating that it was a good supplemental method for HBV screening, diagnosis, and antiviral therapy evaluation. In the future, a large number of clinical samples must be conducted to evaluate and analyze the application of the proposed method for different HBV genotypes, mutants, and courses of HBV infection.

## 5. Conclusions

In summary, a rapid competitive GAM-MNPs-ICA method for hepatitis B preS2Ag detection was developed by adopting GAM-MNPs as a detection probe. A quantitative LOD of 3.6 ng/mL and a qualitive sensitivity of 625 ng/mL were obtained by naked-eye observation within 25 min. The developed GAM-MNPs-ICA had a good stability and reproducibility and a high accuracy. The sensitivity and specificity of the developed GAM-MNPs-ICA method were 93.3% and 90% in clinical serum samples, respectively. It was applicable for the detection of preS2Ag in clinical serum samples and therefore has the potential to be used in large-scale screening for and point-of-care diagnosis of hepatitis B or other infectious diseases.

## Figures and Tables

**Table 1:** Accuracy validation of GAM-MNPs-ICA using the ELISA kit (n = 3).

Spiked PreS2Ag Concentrations (ng/mL)	GAM-MNPs-ICA	ELISA	Relative Error (%)
	PreS2Ag Concentrations (ng/mL)	PreS2Ag Concentrations (ng/mL)	
5	5.91 ± 1.68	5.16 ± 1.34	14.4
50	43.04 ± 6.35	48.27 ± 4.61	10.8
500	456.93 ± 59.76	505.64 ± 82.39	9.6
5000	4517.44 ± 787.50	4551.18 ± 753.04	7.4

**Table 2:** Reproducibility evaluation of GAM-MNPs-ICA (n = 6).

PreS2Ag Concentrations (ng/mL)	Intra-Assay		Inter-Assay	
	*B*/*B*_0_	CV	*B*/*B*_0_	CV
0	1.000 ± 0.021	2.13%	1.009 ± 0.025	2.46%
50	0.928 ± 0.031	3.39%	0.953 ± 0.045	4.74%
500	0.247 ± 0.011	4.45%	0.270 ± 0.030	11.29%
5000	0.079 ± 0.007	9.07%	0.092 ± 0.012	12.72%

**Table 3:** Detection results of the clinical serum sample using GAM-MNPs-ICA and 2 types of ELISA kits (n = 25).

Serological Marker	Method	Positive Results			Negative Results		
		TP	FP	Same Results	TN	FN	Same Results
PreS2Ag	GAM-MNPs-ICA	14	1	12	9	1	9
	ELISA Kit 1	13	0		10	2	
HBsAg	ELISA Kit 2	15	0		10	0	

**Table 4:** Comparison of immunosensors for the quantitative detection of HBV serologic markers.

Target	Method	Label Material	LOD	Detection Time	Quantitative Method	Sample	Reference
HBsAg	ICA	Au@Pt/blue SiNPs	0.13 ng/mL	15 min	Camera/Image J	Spiked FCS	[38]
	ICA	Ultramarine blue particles	0.37 ng/mL	15 min	Camera/Image J	Spiked FCS	[39]
	ICA	Red SiNPs	0.97 ng/mL	10 min	Camera/Image J	Spiked FCS	[40]
	ICA	MNPs	1 ng/mL	20 min	Immuno-chromato Reader	Spiked PBS buffer with 1% BSA	[41]
	ICA	Quantum dots	0.075 ng/mL	15 min	Fluorescence strip reader	Human serum	[42]
	ECA	SPCE	2.1 ng/mL	25 min	EIS	Spiked acetate buffer	[44]
HBcAg	Hydrogel-based QCM biosensor	Hydrogel based on PAAc	0.6 mg/mL	1 h	QCM	Spiked rabbit serum	[45]
PreS1Ag	ECA	AuNPs modified glassy carbon electrode	0.1 pM	80 min	Electrochemical analyser	Human serum	[43]
PreS2Ag	ICA	MNPs	3.6 ng/mL	25 min	Magnetic assay reader	Human serum	This work

Abbreviations: FCS: fetal calf serum; ECA: electrochemical assay; SPCE: screen-printed carbon electrode; EIS: electrochemical impedance spectroscopy; QCM: quartz crystal microbalance; PAAc: poly (acrylic acid).
